# Racial disparities in pedestrian-related injury hospitalizations in the United States

**DOI:** 10.1186/s12889-020-09513-8

**Published:** 2020-09-25

**Authors:** Cara Hamann, Corinne Peek-Asa, Brandon Butcher

**Affiliations:** 1 University of Iowa Injury Prevention Research Center, Iowa City, IA USA; 2 Department of Epidemiology, University of Iowa College of Public Health, Iowa City, IA USA; 3 Department of Occupational and Environmental Health, University of Iowa College of Public Health, 145 N. Riverside Dr, S143 CPHB, Iowa City, IA 52242 USA; 4 Department of Biostatistics, University of Iowa College of Public Health, Iowa City, IA USA

**Keywords:** Walking, Race, Mortality, Health status disparities, Epidemiology

## Abstract

**Background:**

Racial/ethnic disparity has been documented in a wide variety of health outcomes, and environmental components are contributors. For example, food deserts have been tied to obesity rates. Pedestrian injuries are strongly tied to environmental factors, yet no studies have examined racial disparity in pedestrian injury rates. We examine a nationally-representative sample of pedestrian-related hospitalizations in the United States to identify differences in incidence, severity, and cost by race/ethnicity.

**Methods:**

Patients with ICD diagnosis E-codes for pedestrian injuries were drawn from the United States Nationwide Inpatient Sample (2009–2016). Rates were calculated using the United States Census. Descriptive statistics and generalized linear regression were used to examine characteristics (age, sex, severity of illness, mortality rates, hospital admissions, length of stay, total costs) associated with hospitalizations for pedestrian injuries.

**Results:**

The annual average of pedestrian-related deaths exceeded 5000 per year and hospitalizations exceeded 47,000 admissions per year. The burden of injury from pedestrian-related hospitalizations was higher among Black, Hispanic, and Multiracial/Other groups in terms of admission rates, costs per capita, proportion of children injured, and length of stay compared to Whites and Asian or Pacific Islander race/ethnicities. Compared to Whites, hospital admission rates were 1.92 (95% CI: 1.89–1.94) and 1.20 (95% CI: 1.19–1.21) times higher for Multiracial/Other and Blacks, respectively. Costs per capita ($USD) were $6.30, $4.14, and $3.22 for Multiracial/Others, Blacks, and Hispanics, compared to $2.88 and $2.32 for Whites and Asian or Pacific Islanders. Proportion of lengths of stay exceeding one week were larger for Blacks (26.4%), Hispanics (22.6%), Asian or Pacific Islanders (23.1%), and Multiracial/Other (24.1%), compared to Whites (18.6%). Extreme and major loss of function proportions were also highest among Black (34.5%) and lowest among Whites (30.2%).

**Conclusions:**

Results from this study show racial disparities in pedestrian injury hospitalization rates and outcomes, particularly among Black, Hispanic, and Multiracial/Other race/ethnicity groups and support population and system-level approaches to prevention. Access to transportation is an indicator for health disparity, and these results indicate that access to safe transportation also shows inequity by race/ethnicity.

## Background

Racial disparity has been well documented in a wide variety of health outcomes, ranging from infant mortality to asthma to diabetes to cardiovascular disease [1, 2]. Characteristics of the built environment have been identified as an important social determinant of health disparity, as well as disparity related to race/ethnicity [3]. Examples include the relationships between food deserts and nutrition-related metabolic diseases, dilapidated neighborhoods and community violence, air pollution and mortality, and zip code characteristics with cancer and overall mortality [4, 5].

Environmental factors have been strongly tied to pedestrian injuries and deaths in all ages. Environmental risk factors include high speed roads, poor visibility, business density, wide roads, high traffic volume, and absence of pedestrian facilities (e.g. signals, refuge islands) [6–8]. Pedestrian fatalities have increased more than 50% in the past decade and currently account for 17% of all traffic deaths [3]. In 2018, over 180,000 pedestrians visited an emergency department for non-fatal injury treatment [4]. However, the burden of injury from pedestrian hospitalizations by race/ethnicity has not been previously estimated. Based on studies that tie racial inequity in health outcomes to the underlying environmental conditions associated with low resources [5], we hypothesize that non-whites in the US have higher rates of pedestrian injury death and hospitalization per 100,000 population compared to whites. The objective of this study was to identify differences by race/ethnicity for pedestrian mortality rates and to examine incidence, severity, and cost of pedestrian-related injury hospitalizations in a nationally-representative sample.

## Methods

### Study design and data sources

Data for mortality rates were from the Centers for Disease Control and Prevention Web-based Injury Statistics Query and Reporting System (WISQARS) [4] for the years of 2009 to 2016. Data for hospitalizations from 2009 to 2016 were from the US Nationwide Inpatient Sample (NIS). The NIS, a nationally representative database of patient-level all-payer inpatient care, is part of the Healthcare Cost and Utilization Project, maintained by the Agency for Healthcare Research and Quality [9]. The NIS includes sample weights based on hospital characteristics (ownership/control, bed size, teaching status, urban/rural location, and region) that can be used to calculate nationally representative estimates. Hospitalizations with primary or secondary diagnosis external cause of injury e-codes corresponding to pedestrian injuries were included as part of the study sample. Data for rate denominators were from the United States Census total population for each race/ethnic group. Populations for the Census Hispanic categories were aggregated to match the Hispanic definition used by the NIS. Native American was included in the Multiracial/Other category due to low counts.

#### Study variables

The primary dependent variable was total hospital costs. Hospital charges were converted to costs by using the cost-to-charge ratio files provided by the NIS and adjusted to inflation rates for in-hospital care for Q4 of 2016 based on Bureau of Labor Statistics data [10]. Cost per capita by race/ethnicity was computed as the estimated total hospital costs for each race/ethnic group divided by the corresponding total census population estimate of that group. This quantity is interpreted as the dollar amount burden of pedestrian injuries per person in each race/ethnicity group.

We also descriptively examined the following outcomes: length of stay (days), severity of illness, and mortality (died indicated in discharge disposition). Severity of illness was based on the Patient Diagnostic Related Group Severity of Illness variable in the NIS dataset, which includes the following categories: extreme, major, moderate, and minor loss of function.

The primary independent variable was race/ethnicity (Black, Hispanic, Asian or Pacific Islander, White, and Multiracial/Other). Native Americans and anyone identifying as multi-racial were included in the Multiracial/Other category. Interactions between age, sex, and race were examined and it was determined by QIC that a model with only main effects for these variables best fit the data.

#### Analysis

National estimates of frequencies and proportions were calculated for all patient and hospital characteristics of interest (age, sex, severity of illness, mortality rates, hospital admissions, length of stay and total costs) by race/ethnic group. Generalized Estimating Equations (GEE) based on the gamma distribution with an exchangeable working correlation matrix were used to estimate hospital costs per admission adjusted for patient and hospital level characteristics (age, sex, length of stay, severity of illness, income, payer source, indicator for ICD-9 vs. ICD-10, hospital region, & hospital location/teaching status). All estimates incorporated NIS sampling weights and the NIS sampling design to provide nationally representative estimates and standard errors. SAS 9.4 was used for all statistical analyses.

## Results

For the years 2009 to 2016, there were 40,576 deaths and 376,417 total estimated hospitalizations for pedestrian-related injuries in the United States. Annually, that translates to an average of more than 47,000 pedestrian-related hospitalizations in the United States, accounting for an estimated $1.13 billion in total hospital costs per year. The overall age-adjusted rate of pedestrian-related deaths increased from 1.31 in 2009 to 1.88 per 100,000 population in 2016 [4], while the estimated age-adjusted rate of hospitalizations decreased from 15.97 in 2009 to 12.40 per 100,000 population in 2016.

**Fig. 1:**
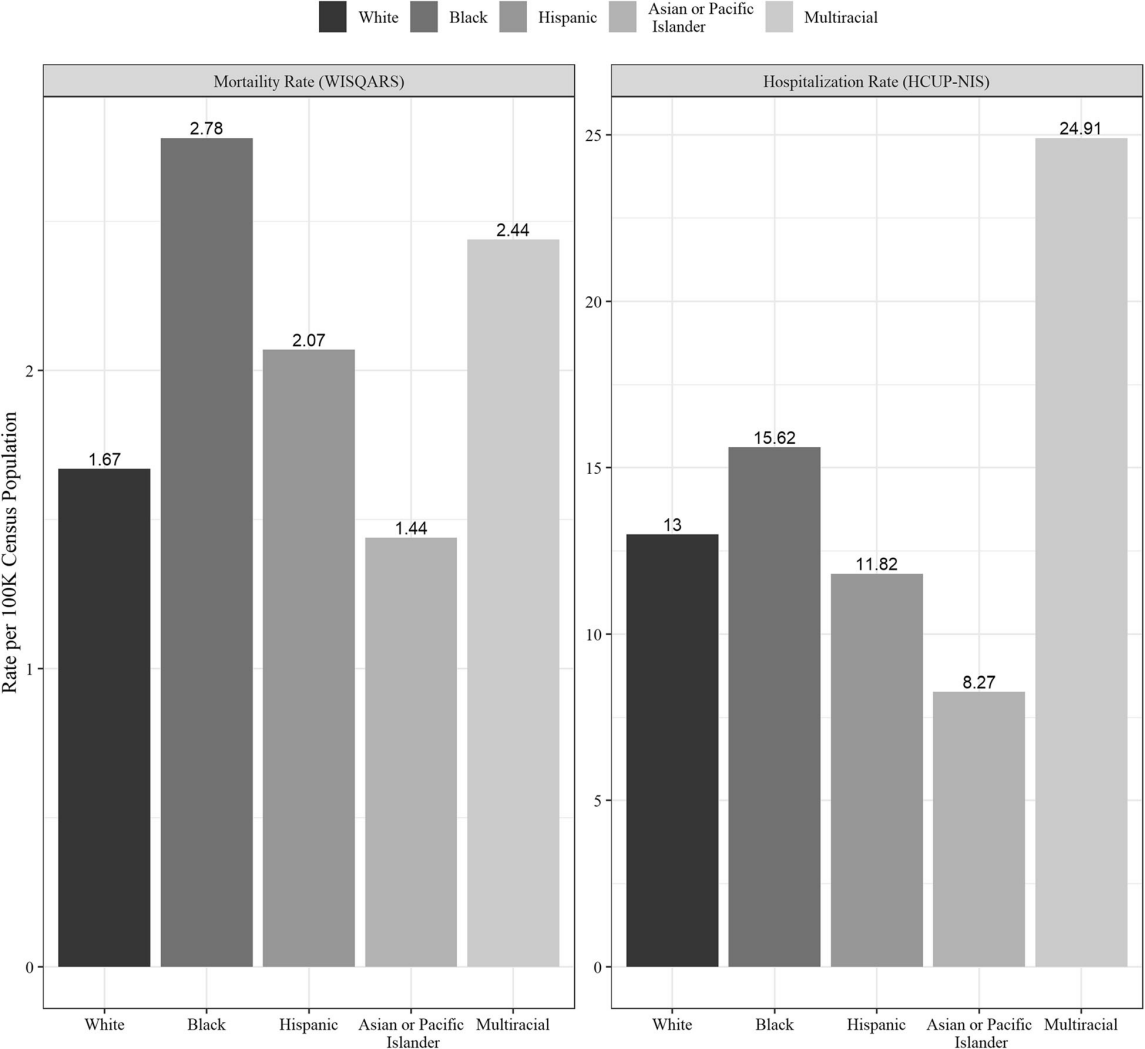
Mortality rate of pedestrian injuries per 100,000 census population from WISQARS and Hospitalization rate per 100,000 census population estimated from HCUP-NIS, 2009–2016

Pedestrian mortality rates per 100,000 population (Fig. 1) were highest for Multiracial/Others (2.44) and Blacks (2.78). Rates of hospital admissions (Fig. 1) were higher for Blacks (15.6) and Multiracial/Other (24.9) compared to Whites (13.0), Hispanics (11.8), and Asian or Pacific Islander (8.3) race/ethnicities.

**Fig. 2:**
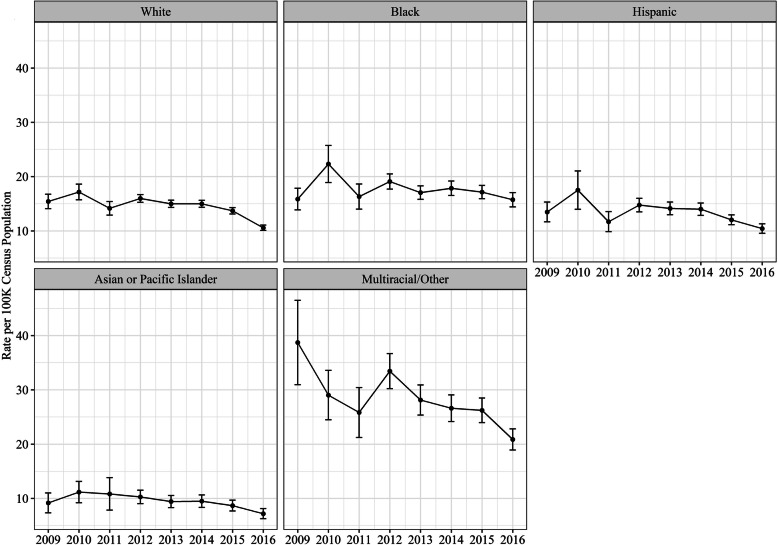
Annual hospitalization admission rates by race estimated from HCUP-NIS, 2009–2016

Compared to Whites, the rate of hospital admissions were 1.92 and 1.20 times higher for Multiracial/Other and Blacks, respectively (Fig. 2). Whereas, the rate of hospital admissions were 36% (Rate Ratio = 0.64, 95% CI = 0.63–0.65) and 9% (Rate Ratio = 0.91, 95% CI = 0.90–0.92) lower for Asian or Pacific Islander and Hispanics, respectively.

Black, Hispanic, and Multiracial/Other groups had the highest costs per capita (Table 1). Pedestrian hospitalizations carry a burden of $4.14, $3.22, and $6.30 per capita for Blacks, Hispanics, and Multiracial/Others, compared to $2.88 and $2.32 for Whites and Asian or Pacific Islanders. Higher proportions of children and young people (ages 24 and under) were injured among Black (32.5%), Hispanic (35.8%), and Multiracial/Other (30.7%) groups, compared to White (21.9%) and Asian or Pacific Islander (22.8%). However, these age patterns are as expected, as young people make up a greater proportion of the population among those race/ethnic groups [11]. Compared to Whites, all other race/ethnicities had more lengths of stay exceeding one week. Extreme and major loss of function proportions were also higher among Blacks compared to other race/ethnicities.

**Table 1:** Descriptive statistics of hospital admissions by race/ethnicity, United States, 2009–2016

Variable	White	Black	Hispanic	Asian or	Multiracial/Other	Overall
				Pacific Islander		
Weighted Number (Percent) of Hospital Admissions (n, %)*	231,245 (61.4)	55,213 (14.7)	58,057 (15.4)	12,656 (3.4)	19,246 (5.1)	376,417
***Hospital Admission Rate Ratios (95% CI)***	Reference	1.20 (1.19–1.21)	0.91 (0.90–0.92)	0.64 (0.63–0.65)	1.92 (1.89–1.94)	–
Age (n, %)*						
< 5	3102 (1.3)	1668 (3.0)	2674 (4.6)	292 (2.3)	599 (3.1)	8336 (2.2)
5–14	18,895 (8.2)	6807 (12.3)	7987 (13.8)	1091 (8.6)	2277 (11.8)	37,057 (9.8)
15–24	28,766 (12.4)	9470 (17.2)	10,119 (17.4)	1503 (11.9)	3044 (15.8)	52,902 (14.1)
25–44	51,496 (22.3)	14,861 (26.9)	16,115 (27.8)	2663 (21.0)	5315 (27.6)	90,450 (24.0)
45–64	81,632 (35.3)	17,720 (32.1)	14,260 (24.6)	3618 (28.6)	5338 (27.7)	122,567 (32.6)
65+	47,354 (20.5)	4688 (8.5)	6902 (11.9)	3489 (27.6)	2672 (13.9)	65,105 (17.3)
Sex (n, %)*						
Female	72,208 (31.2)	17,609 (31.9)	16,955 (29.2)	5923 (46.8)	6345 (33.0)	119,041 (31.6)
Male	159,037 (68.8)	37,604 (68.1)	41,101 (70.8)	6734 (53.2)	12,900 (67.0)	257,376 (68.4)
Length of Stay (days) (n, %)*						
0	8340 (3.6)	2161 (3.9)	2390 (4.1)	524 (4.1)	809 (4.2)	14,224 (3.8)
3–4	118,787 (51.4)	24,681 (44.7)	29,300 (50.5)	6186 (48.9)	9345 (48.6)	188,298 (50.0)
4–7	61,206 (26.5)	13,795 (25.0)	13,249 (22.8)	3025 (23.9)	4456 (23.2)	95,731 (25.4)
> 7	42,912 (18.6)	14,577 (26.4)	13,119 (22.6)	2922 (23.1)	4636 (24.1)	78,165 (20.8)
All Patient Diagnostic Related Group Severity of Illness (n, %)*						
Extreme loss of function	21,182 (9.2)	6781 (12.3)	5951 (10.3)	1361 (10.8)	2278 (11.8)	37,554 (10.0)
Major loss of function	48,608 (21.0)	12,279 (22.2)	11,712 (20.2)	2591 (20.5)	3872 (20.1)	79,062 (21.0)
Moderate loss of function	97,679 (42.2)	20,995 (38.0)	21,931 (37.8)	4913 (38.8)	7403 (38.5)	152,920 (40.6)
Minor loss of function	63,777 (27.6)	15,159 (27.5)	18,463 (31.8)	3791 (30.0)	5693 (29.6)	106,882 (28.4)
Died (n, %)*						
No	225,273 (97.4)	53,671 (97.2)	56,093 (96.6)	12,092 (95.5)	18,509 (96.2)	365,638 (97.1)
Yes	5973 (2.6)	1542 (2.8)	1964 (3.4)	564 (4.5)	737 (3.8)	10,780 (2.9)
Discharge disposition (n, %)*						
Other	84,816 (36.7)	19,470 (35.3)	15,929 (27.4)	4980 (39.4)	6626 (34.4)	131,821 (35.0)
Routine	146,429 (63.3)	35,744 (64.7)	42,127 (72.6)	7676 (60.6)	12,620 (65.6)	244,596 (65.0)
Payer Source (n, %)*						
Government	73,462 (31.8)	22,574 (40.9)	22,902 (39.4)	3858 (30.5)	5846 (30.4)	128,642 (34.2)
Other	18,594 (8.0)	4787 (8.7)	6004 (10.3)	813 (6.4)	1560 (8.1)	31,757 (8.4)
Private, Including HMO	115,310 (49.9)	19,069 (34.5)	18,903 (32.6)	6807 (53.8)	9280 (48.2)	34,292 (45.0)
Self-Pay	2559 (13.3)	8784 (15.9)	10,248 (17.7)	1179 (9.3)	2559 (13.3)	9471 (12.4)
***Costs*** **per capita*, $USD (Mean ± SD)***	2.88 ± 0.10	4.14 ± 0.19	3.22 ± 0.22	2.32 ± 0.25	6.30 ± 0.35	3.16 ± 0.11

*Chi-square *p*-value < 0.0001

Compared to Whites, all other race/ethnic groups had higher adjusted hospital costs (Table 2). Furthermore, adjusted hospital costs by each payer source (government, private, other) were lower for Whites compared to all other combinations of race and payer source, with the exception of self-pay.

**Table 2:** Adjusted hospital costs per admission (95% CI) by race/ethnicity and by race/ethnicity by payer source

Variable	White	Black	Hispanic	Asian or Pacific Islander	Multiracial/Other	Overall
***All Payer Sources***	17,300 (17,000 - 17,500)	19,000 (18,700 - 19,500)	18,500 (18,000 - 19,000)	19,600 (18,900 - 20,400)	18,800 (18,200 - 19,400)	17,900 (17,700 - 18,100)
***Payer Source***						
Government	18,200 (17,900 - 18,500)	20,170 (19,500 - 20,800)	18,920 (18,300 - 19,600)	24,000 (22,500 - 25,600)	20,400 (19,300 - 21,500)	18,900 (18,600 - 19,200)
Other	19,300 (18,700 - 19,900)	20,800 (19,500 - 22,100)	21,800 (20,000 - 23,700)	19,500 (17,200-22, 100)	20,600 (19,300 - 21,500)	20,000 (19,400 - 20,600)
Private, Including HMO	16,500 (16,300 - 16,800)	18,700 (18,100 - 19,300)	18,000 (17,300 - 18,600)	17,600 (16,700 - 18,600)	18,300 (17,500 - 19,100)	17,100 (16,800 - 17,300)
Self-Pay	16,400 (16,000 - 16,900)	16,600 (15,800 - 17,400)	16,900 (16,100 - 17,700)	18,800 (17,000 - 20,800)	16,200 (15,000 - 17,500)	16,600 (16,200 - 17,000)

## Discussion

Racial disparities in pedestrian deaths and injuries were present in both hospitalization admission rates and outcomes. Mortality rates among Black, Hispanic, and Multiracial/Other and hospitalization admission rates among Black and Multiracial/Other race/ethnicity groups were particularly high compared to White and Asian or Pacific Islanders. The Black, Hispanic, and Multiracial/Other race/ethnic groups carry a larger burden of injury with increased hospital costs, cost per capita, severity of illness, and lengths of stay.

Results from this study align with a large body of literature related to racial disparities and health outcomes, which has consistently shown length of stay and cost disparities among Black, Hispanic, and Multiracial/Other race/ethnicities, compared to Whites [12, 13]. A study of nearly 50,000 U.S. pedestrian deaths using CDC annual mortality data (1999–2015) found significant disparities in death rates by racial/ethnic groups, with higher rates among Blacks, Latinos, and Native Americans, compared to Whites [14], whereas our results showed similar racial/ethnic disparities in injury severity and burden of injury among pedestrian hospitalizations with higher costs per capita, longer lengths of stay, and increased mortality among Blacks, Hispanics, and Multiracial/Others, compared to Whites. Our results also align with previous research that has established links between race and inequities in safety and accessibility of transportation, including walking, [15] and neighborhood social inequities, traffic volumes, road design, and road traffic injuries [6].

Accurate pedestrian exposure data by race/ethnicity are not available but are important for understanding the mechanisms behind these disparities. For example, differential exposure to walking (i.e., time spent walking and exposed to traffic) and risky pedestrian environments (e.g., higher traffic volumes, lack of pedestrian facilities) may contribute to these differences.

However, the specific mechanisms behind these disparities are complex, as evidenced within the existing literature. A UK-based study found higher rates of child pedestrian injuries among Blacks, regardless of area level deprivation, but found that area deprivation was an important factor among Whites and Asians with higher injury rates correlating with higher deprivation [16]. Those results suggest neither race/ethnicity or area deprivation (e.g, built environment, socio-economic factors) alone can adequately explain race/ethnicity differences. A study of US fatality data found higher rates of pedestrian and bicyclist fatalities among neighborhoods with high proportions of Black and Hispanic residents as compared to predominant White or Asian neighborhoods [17]. However, they also found predominant Black and Hispanic neighborhoods to be safer, overall, in terms of road traffic fatality rates compared to predominant non-Hispanic White neighborhoods [17]. Another possible factor includes racial bias, which has been found in driver yielding behavior at crosswalks [18–20].

Access to transportation is a recognized social determinant of health, but disparities and inequities in transportation access and safety have largely been overlooked, despite having important ramifications related to health and well-being (access to food, jobs, etc.) [21]. The persistence and variety of health outcomes which demonstrate racial disparity indicate that the focus of prevention would best be placed at the population-level and on systems-based factors, rather than at the individual level or on issues related to race/ethnicity directly [22, 23]. These factors include economic, built environment, and transportation policy changes and interventions. There is also evidence that focusing on the social environment and social determinants of health with a broad range of interventions, such as early childhood development programs, rental assistance programs, and de-concentration of public housing, can reduce racial disparities in health [24, 25].

Specific to reducing the burden from pedestrian injuries, interventions may include amending policies to resolve inequities in transportation access, improvements to the built environment to include pedestrian facilities (e.g., crosswalks, pedestrian signals), safety culture campaigns focused on geospatial areas at high risk, and geographic equity in road safety funding for both new projects and maintenance (e.g., repair and maintenance of roads and sidewalks).

This study is based on a nationally-weighted sample of hospital admissions, which should be strongly representative of the US population. Limitations include potential miscoding of pedestrian injuries, which can happen when the role of the injured person in the crash is not known at hospital admission, or miscoding of race and ethnicity. Lack of information about differential pedestrian exposure/activity by race/ethnicity, and particularly how built environments differ by race/ethnicity, are a limitation in making recommendations about prevention measures based on these findings.

Results from this study show that the burden of injury from pedestrian injuries is higher among non-Whites, which has important implications in development of prevention and intervention approaches as we work to combat the rising pedestrian fatality and injury toll in the United States. Detailed pedestrian exposure data are also needed to further examine possible mechanisms for these disparities.

## Conclusions

This study adds to growing evidence of the ties between the physical environment and health. The persistence of racial disparities in health outcomes indicates priority for prevention approaches at the population and systems-level, as opposed to individual-level approaches. Thus, access to safe transportation should be integrated into transportation policy and infrastructure decisions.

## Data Availability

The National Inpatient Sample is available through the Healthcare Utilization Project of the Agency for Healthcare Research and Quality: https://www.hcup-us.ahrq.gov/nisoverview.jsp.
